# Treatment of disabling headache with greater occipital nerve injections in a large population of childhood and adolescent patients: a service evaluation

**DOI:** 10.1186/s10194-018-0835-5

**Published:** 2018-01-16

**Authors:** Francesca Puledda, Peter J. Goadsby, Prab Prabhakar

**Affiliations:** 1 Headache Group, Department of Basic and Clinical Neuroscience, King’s College London, and NIHR-Wellcome Trust King’s Clinical Research Facility, Wellcome Foundation Building, King’s College Hospital, London, SE5 9PJ UK; 2 Department of Paediatric Neurology, Great Ormond Hospital for Children NHS Foundation Trust, London, UK

**Keywords:** Pediatric headache, Greater occipital nerve injection, Chronic migraine, Trigeminal autonomic cephalalgia, Cluster headache, New daily persistent headache

## Abstract

**Background:**

Pediatric headache disorders can be extremely disabling, with marked reduction in the quality of life of children and their carers. Evidenced-based options for the treatment of primary headache disorders with preventive medication is limited and clinical outcomes are often unsatisfactory. Greater occipital nerve injections represent a rapid and well-tolerated therapeutic option, which is widely used in clinical practice in adults, and has previously shown a good outcome in a pediatric population.

**Methods:**

This service evaluation reviewed greater occipital nerve injections performed unilaterally with 30 mg 1% lidocaine and 40 mg methylprednisolone, to treat disabling headache disorders in children and adolescents.

**Results:**

We analyzed a total of 159 patients who received 380 injections. Of the population, 79% had chronic migraine, 14% new daily persistent headache, 4% a trigeminal autonomic cephalalgia, 3% secondary headache and one patient had chronic tension-type headache. An improvement after injection was seen in 66% (*n* = 105) of subjects, lasting on average 9 ± 4 weeks. Improvement was seen in 68% of patients with chronic migraine, 67% with a trigeminal autonomic cephalalgia and 59% with new daily persistent headache. Side effects were reported in 8% and were mild and transient.

Older age, female gender, chronic migraine, increased number of past preventive use, medication overuse and developing side effects were all associated with an increased likelihood of positive treatment outcome.

**Conclusions:**

This large single centre service evaluation confirms that unilateral injection of the greater occipital nerve is a safe, rapid-onset and effective treatment strategy in disabling headache disorders in children, with a range of diagnoses and severity of the condition, and with minimal side effects.

## Background

Headache is the most common manifestation of pain in childhood and can severely impact the health, functional status and quality of life of pediatric patients [1]. In particular, headache disorders with high frequency of attacks in children and adolescents can be extremely disabling. The one-year prevalence of migraine in children between the ages of 5 and 15 years ranges around 10% depending on the study [2], while chronic migraine affects between 0.8% and 1.7% of adolescents [3] and up to 1.7% of subjects in pediatric age groups [4]. The prevalence is up to three times higher in females [5, 6]. When primary headache disorders manifest daily they may be misdiagnosed and very often present co-morbidities such as anxiety, mood disorders, sleep issues and other pain syndromes [7], which require themselves to be addressed.

For all these reasons, early management of disabling headache as early in its course as possible is highly desirable and should involve several strategies, including prevention. In this context, therapy can be extremely challenging, with physicians often facing difficulty in finding rapid and sustained treatment options with a demonstrated efficacy and tolerability in the pediatric population. Recent evidence confirms this, showing that common preventive treatments used in adult migraineurs may not be as effective in pediatric patients [8].

Infiltration of the area around the greater occipital nerve with a mixture of local anesthetic and corticosteroids is a well-established therapy for primary headache prevention in adults [9, 10] that has been recently reported in children to have excellent results [11]. Greater occipital nerve (GON) injections have shown to provide a quick onset of therapeutic response, which is also sustained [12], while avoiding the common side effects of classic migraine preventives or more invasive treatments [13]. The mechanism of action is linked to the anatomical overlap between spinal afferents providing sensory innervation from the C2 occipital region and trigeminal afferents at the trigeminocervical complex, a complex brain area involved in the pathophysiology of primary headache disorders [14].

Our objective was to determine the efficacy and safety of greater occipital nerve injections in a large population of paediatric headache patients.

## Methods

### Study population and design

We performed a service evaluation of the Specialist Headache Centre at Great Ormond Street Hospital for Children and required no Research Ethics Committee approval (http://www.hra-decisiontools.org.uk/research/). A retrospective chart review was performed on all letters and clinical correspondence for patients who received a greater occipital nerve injection between 2009 and 2016. The population comprised children and adolescents seen within the Headache Centre by authors (PP and PJG), always in the presence of either a parent or guardian. Whenever possible, patient histories were taken directly from the children themselves. Headache diagnoses were defined according to ICHD-III-beta [15].

### Data collection

Retrospective data abstraction was performed by one of the authors (FP). For each patient who received a GON injection, information on age (measured as a continuous variable), gender, headache diagnosis, date of first visit, time from first visit to injection, site of injection, past and current medication, effects of injection and eventual follow-up treatment was collected using a standardized pro forma.

### Greater occipital nerve injections

Infiltrations of the region of the greater occipital nerve were always performed unilaterally and consisted of 30 mg 1% lidocaine and 40 mg methylprednisolone acetate. The clinician palpated over the greater occipital nerves and injected the side that was most tender. GON injection was administered 1–2 cm below the midpoint between the occipital tubercle and mastoid process in all patients. The injection site was then massaged to spread the solution.

For the purpose of data collection, we included both first time and repeat injections. The primary outcome of ‘improvement’ from the injection was defined as either a significant, more than one third, decrease in headache frequency or intensity or by a documented headache improvement in the clinical notes.

### Data analysis

All data was tabulated in Excel and analysed using IBM SPSS Statistics 22. *P* < 0.05 was considered significant. Descriptive analysis of numeric variables was performed using measures of central tendency and dispersion. For categorical variables, distribution was described as percentages. Differences in frequencies were examined using Chi-square analysis. A binary logistic regression analysis was performed in order to determine the effect of several predictor variables on the dichotomous primary outcome measure of improvement.

## Results

Two hundred and five patients received at least one greater occipital nerve block, for a total of 458 injections. All patients who were offered an injection had a disabling headache condition.

### Clinical phenotype

Follow-up data for the first injection was available for 78% of patients (*n* = 159), who had a total 380 injections. Of these, 159 patients with follow-up, 79% (*n* = 126) had chronic migraine, 15% (*n* = 24) with aura, 14% (*n* = 22) new daily persistent headache (NDPH), 4% (*n* = 6) a trigeminal autonomic cephalalgia (TAC), 3% (*n* = 4) a form of secondary headache and one patient had chronic tension-type headache. Demographic characteristics of the population are summarized in Table 1.

**Table 1:** Demographic characteristics of 159 patients treated with greater occipital nerve injection

Total patients	*n =* 159	
Mean age (years ± SD; range)	15 ± 2;	range 8–18 years
Female: male (*n*:*n*); %	108:50;	68% - 32%
Headache diagnosis (*n*; %)		
Chronic migraine without aura (CM)	102	64%
Chronic migraine with aura (CMwA)	24	15%
* Total chronic migraine*	126	79%
NDPH	22	14%
CH	3	2%
HC	1	0.6%
SUNCT/SUNA	2	1%
* Total TAC*	6	4%
TTH	1	0.6%
Secondary HA	4	3%
Years of headache to 1st injection (years ± SD; range)	4.5 ± 3.2;	range 0–12 years
Past medication (mean ± SD)	2.1 ± 1.5	range 0–5
Patients on preventive medication at time of injection (*n*)	115	
Diagnosis of medication overuse at time of injection (*n*; %)	36;	23%

Medication overuse was present in 23% (*n* = 36) of subjects.

The mean age was 15 ± 2 with a range between 8 and 18 years. *n* = 12 subjects fell within the 8–12 age range, *n* = 78 in the 13–15 range and *n* = 69 in the 16–18 range.

Female to male ratio was 2.2:1 Mean number of headache years was 4 ± 3 and on average patients had tried at least two previous preventives with a range between 0 and 5.

### Efficacy

A benefit from the GON injection was seen in 66% (*n* = 105) of subjects and this was significant respect to the number of patients who had no effect (*p* < 0.001; see Table 2). The mean duration of improvement was 9 ± 4 weeks with a minimum of 3 weeks (in *n* = 5 patients); the remaining one-hundred patients had an improvement which lasted for more than 3 weeks.

**Table 2:** Effects of first greater occipital nerve injection in 159 chronic headache patients with available follow-up

Improvement from injection	*n* = 105 *P* < 0.001	66%
Duration of improvement (weeks)	9.3 ± 4.3	
Benefit per HA diagnosis (*n*; %)		
CM	70	69%
CMwA	15	63%
* Total migraine*	85	68%
NDPH	13	59%
CH	3	100%
HC	0	0%
SUNCT/SUNA	1	50%
* Total TAC*	4	67%
TTH	1	100%
Secondary HA	2	50%
Sustained HA freedom (>3 weeks)	17	16%
CM	11	16%
CMwA	2 (one brainstem)	13%
* Total migraine*	13	*10% of total migraine*
NDPH	2	15%, *9% of total NDPH*
CH	1	33%
SUNCT	1	100%
* Total TAC*	2	*33% of total TAC*
Side effects	13	8%
Worsened HA int/freq for up to 5 weeks	6 (with no improvement)	
Worsened HA int/freq for up to 10 days	5 (with improvement)	
Soreness	1	
Reaction to sedative	1	

Improvement was seen in 68% of the chronic migraine population (*n* = 85) and 59% (*n* = 13) in the NDPH subgroup. Four of the six patients (67%) with trigeminal autonomic cephalalgias had benefit from the injection. We performed a Chi-square analysis to find differences in frequencies between headache diagnosis and the primary outcome measure; having excluded tension-type headache and secondary headache cases for low numbers, there was no significant difference in improvement between migraine, NDPH and trigeminal autonomic cephalalgias. Sustained headache freedom of more than 3 weeks was seen in 17 patients, of which the majority had chronic migraine.

A total of ninety-nine patients received subsequent injections, and fifty-one of these went on to have more than two. These were given at variable intervals, with a minimum twelve-week gap in between injections. Of patients who had subsequent injections *n* = 15 were headache free and *n* = 67 had a general benefit after treatment.

### Side effects

Side effects were reported in thirteen patients: eleven subjects had a headache worsening, however in five of these a beneficial effect followed after a maximum ten-day interval. One patient reported soreness at the site of injection and one had an allergic reaction, possibly to the sedative that had to be used during the procedure.

### Modelling

A binary logistic regression model was created in order to examine the effect of age, gender, medication overuse, headache diagnosis and frequency, years of disease, number of past preventives used and presence of side effects, on the primary outcome measure of improvement for the first GON injection. Results of the regression analysis are shown in Table 3. In summary, no specific variable was responsible for significantly predicting a positive outcome, although a diagnosis of migraine and a trigeminal autonomic cephalalgia increased the odds of having an improvement from the injection by 4 and 3 times, respectively. Each increase in age by year made the odds of improvement 1.2 times more likely (95% CI 0.8–1.8) as well as each increase in discreet number of past medications used and being a female. Having a diagnosis of medication overuse and having developed side effects to the injection were also associated with an increase likelihood of having an improvement after the treatment. The predictor variables inserted in the model were subsequently removed one at a time, causing no significant change in results and therefore allowing to exclude any confounding effect of single variables on the model.

**Table 3:** Results of binary logistic regression analysis for likelihood of improvement from greater occipital nerve injection. Variables in italics were associated with negative beta values and therefore a decreased likelihood of the outcome

Predictor Variable	*p* value	Odds ratio	95% Confidence Interval
Age (measured in years)	0.38	1.2	0.8–1.8
Gender (female)	0.74	1.2	0.4–3.6
Chronic migraine diagnosis	0.32	4.0	0.3–62.2
TAC diagnosis	0.45	3.0	0.2–50.6
Medication overuse	0.89	1.1	0.4–3.1
*Headache frequency (days)*	*0.47*	*1.0*	*0.9–1.1*
*Years of headache*	*0.58*	*1.0*	*0.8–1.1*
Number of past preventives	0.46	1.2	0.8–1.6
*Time from diagnosis to injection (weeks)*	*0.18*	*1.0*	*0.9–1.0*
Side effects	0.53	1.8	0.3–11.0

## Discussion

Disabling headaches can be extremely challenging for both patients and clinicians [1], especially given that the armamentarium of available strategies presents several caveats, making adherence to treatment generally quite low [16]. Our service evaluation suggests greater occipital nerve injections can be useful across a range of primary headache disorders, offering an effective, low-risk procedure.

The majority of patients improved after the first GON injection. This is in accordance with previous observations in adults [12] and children [11]. Interestingly, our study found no significant difference with regards to response to treatment in different headache phenotypes, even if we observed an increased likelihood of response in migraineurs and patients with TACs. It must be noted however that the number trigeminal autonomic cephalalgia patients was quite low (*n* = 6). It is noteworthy that the clinical trial literature for GON injections is stronger in cluster headache [10, 17] than migraine [18]. Our results are quite encouraging, as it shows that GON injections can be used to treat a variety of complex headache problems regardless of the underlying diagnosis.

Reported studies have shown a great variability exists with regards to clinical practice and approach to this technique [19, 20], and more effort needs to be expended to standardize the general procedure, as well as in obtaining data from controlled trials in different population groups. Even though GON injections have been used for several years in headache practice [21] and other paediatric conditions [22], generally the paediatric headache literature is quite limited, with only one systematic study being performed recently in a North-American headache centre [11]. To the best of our knowledge, this is the first large survey on the practice of greater occipital nerve injections in a European paediatric population.

Treatment response was generally prolonged with 16% of patients remaining headache free for more than 3 weeks. This, even if not comparable with that of regular pharmacological preventives, represents a positive result especially considering that GON injections are a one-off intervention. In this context their use as a transitional therapy can be encouraging for patients. Unfortunately, data on onset of effect from the procedure was not available for all patients, therefore we cannot comment on time of therapeutic response, even though past studies have shown this to be quite rapid [11].

GON infiltrations showed a very high tolerability in our population, with the most common side effect being an initial worsening of the pre-existing headache condition, which lasted up to 5 weeks in the worst case. However, almost half of patients who experienced this side effect subsequently responded to treatment with a significant improvement.

A repeat injection was performed in more than half the patients who had an initial improvement, a large majority of which continued to report a benefit from treatment.

Results from binary logistic regression analysis suggested no significant effect of several clinical parameters in predicting a positive outcome to treatment, although certain conditions, such as chronic migraine, were linked to an increased likelihood of developing an effect from the injection. This result, however, does not exclude that an effective response could be achieved in new daily persistent headache - the second most common chronic headache diagnosis in our population - or other headache phenotypes, as seen by the Chi-square sub analysis.

It is interesting to note that conditions normally associated with a more severe clinical picture, such as an increased number of headache preventive medications used in the past and a diagnosis of medication overuse, not only did not predict a poorer response to treatment but on the contrary were linked with a higher likelihood of improvement. This is also true for the developing of injection side effects, such as a worsening of headache conditions, which in almost half the cases showed to revert to a substantial improvement after a few days. It is therefore possible to infer that greater occipital nerve injections are a valid therapeutic approach even in refractory headache patients who would normally be bound to fail normal preventives [23]; patients should also be informed that side effects are not to be considered as a marker of poor outcome. Finally, our data showed that older children and female patients are generally more likely to have a response to treatment and this is in line with previous studies.

### Limitations

An important limitation of this study is that being compiled from an open label series, a placebo effect cannot be excluded. It is worth noting this is a relatively invasive procedure and the proportion of patients showing a placebo response in headache studies is well established [24]. However, the large number of patients analysed does offer insight on the advantages of this treatment with respect to more classic preventive therapies. In the future, research should focus on performing large randomized controlled studies in paediatric subjects to establish how objectively effective this approach may be.

## Conclusions

Greater occipital nerve injections are a safe, effective and useful strategy for disabling primary headache disorders in children. They appear especially beneficial in patients with migraine and trigeminal autonomic cephalalgias, although phenotypical diagnosis is not an apparent limitation to its effect. Presence of side effects and refractory headache does not predict poor treatment outcome. In the clinical approach to the treatment of chronic primary headache disorders in a paediatric setting, GON injections should be considered as first line management alongside the classic medications, which are often more side-effect prone.

## Abbreviations

CH: Cluster headache
CM: Chronic migraine without aura
CMwA: Chronic migraine with aura
GON: Greater occipital nerve
HA: Headache
HC: Hemicrania continua
ICHD-III-beta: International classification of headache disorders 3 beta
NDPH: New Daily Persistent Headache
SUNA: Short-lasting unilateral neuralgiform headache attacks with cranial autonomic symptoms
SUNCT: Short lasting unilateral neuralgiform headache attacks with conjunctival injection and tearing
TAC: Trigeminal autonomic cephalalgia
TTH: Tension-type headache
